# Application of stentless mitral valve to surgery in infective endocarditis: Partial Normo valve repair

**DOI:** 10.1016/j.xjtc.2025.05.007

**Published:** 2025-05-30

**Authors:** Tomoya Uchimuro, Minoru Tabata, Joji Ito, Kaito Masuda, Yuki Matsui, Hitoshi Kasegawa, Shuichiro Takanashi

**Affiliations:** a Department of Cardiac Surgery, Kawasaki Saiwai Hospital, Kawasaki, Japan; b Department of Cardiovascular Surgery, Juntendo University Hospital, Tokyo, Japan; c Department of Cardiovascular Surgery, Tokyo Bay Urayasu Ichikawa Medical Center, Chiba, Japan; d Department of Cardiac Surgery, Cardiovascular Institute Hospital, Tokyo, Japan

**Figure:**
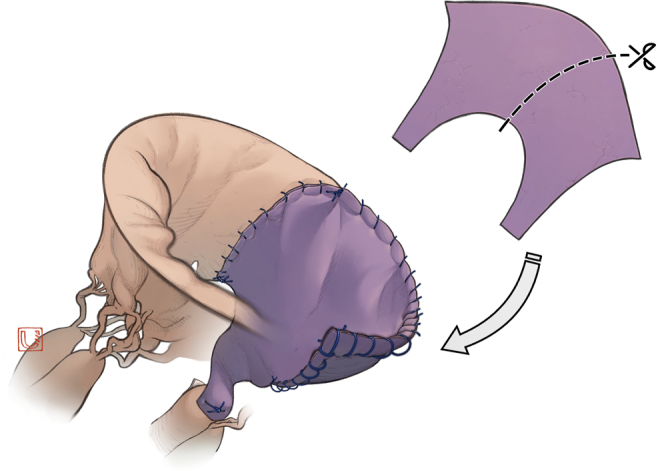
Partial Normo valves can reconstruct a wide range of infected lesions.

**Central Message**

Partial Normo valve repair enabled successful MV repair in extensive IE involving the commissure.

Methods for mitral valve (MV) repair for infective endocarditis (IE) are well established.^1^^,^^2^ However, the extent of infection varies between patients. When infection involves the commissure, we have partially used the Normo valve developed by Kasegawa and colleagues.^3^^,^^4^ We present partial Normo valve repair, which we developed for reconstructing an area including the commissure. Between April 2019 and September 2024, 6 patients were treated using this at our institutions. Patient data are presented in Table 1.

**Table 1:** Patients’ data

Variables	N = 6
Male	3
Age, y	44.8 (26-68)
Location of infection	
A3 + PC + P3	3
A2 + A3 + PC	2
AC + P1	1
Prosthetic ring annuloplasty	5
Crossclamp time, min	153 (111-197)
Cardiopulmonary bypass time, min	212 (164-284)
MR before discharge	
mild or less	6
MR at last follow-up	
mild or less	5
moderate	1
Follow- up period, mo	23 (6-37)

*MR,* Mitral regurgitation.

## Surgical Technique

**Figure 1:**
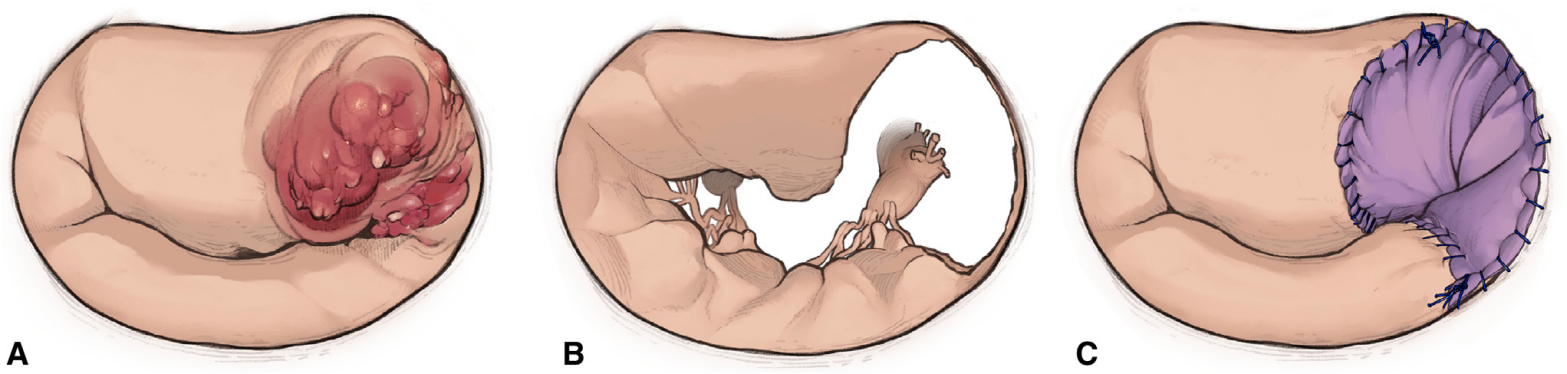
A, Infection involves posterior commissure. B, Infected tissues are resected. C, Resected area is repaired.

**Figure 2:**
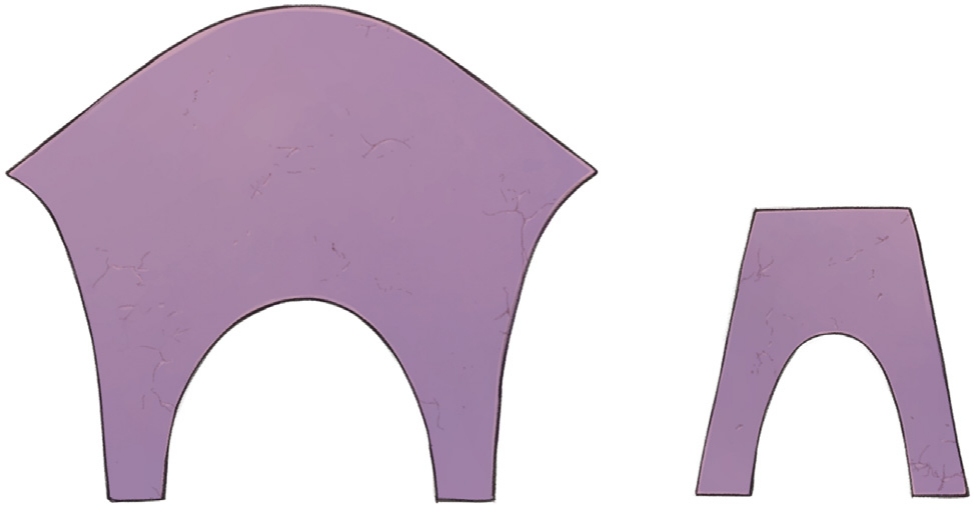
Normo valve template consists of a larger and a smaller part.

**Figure 3:**
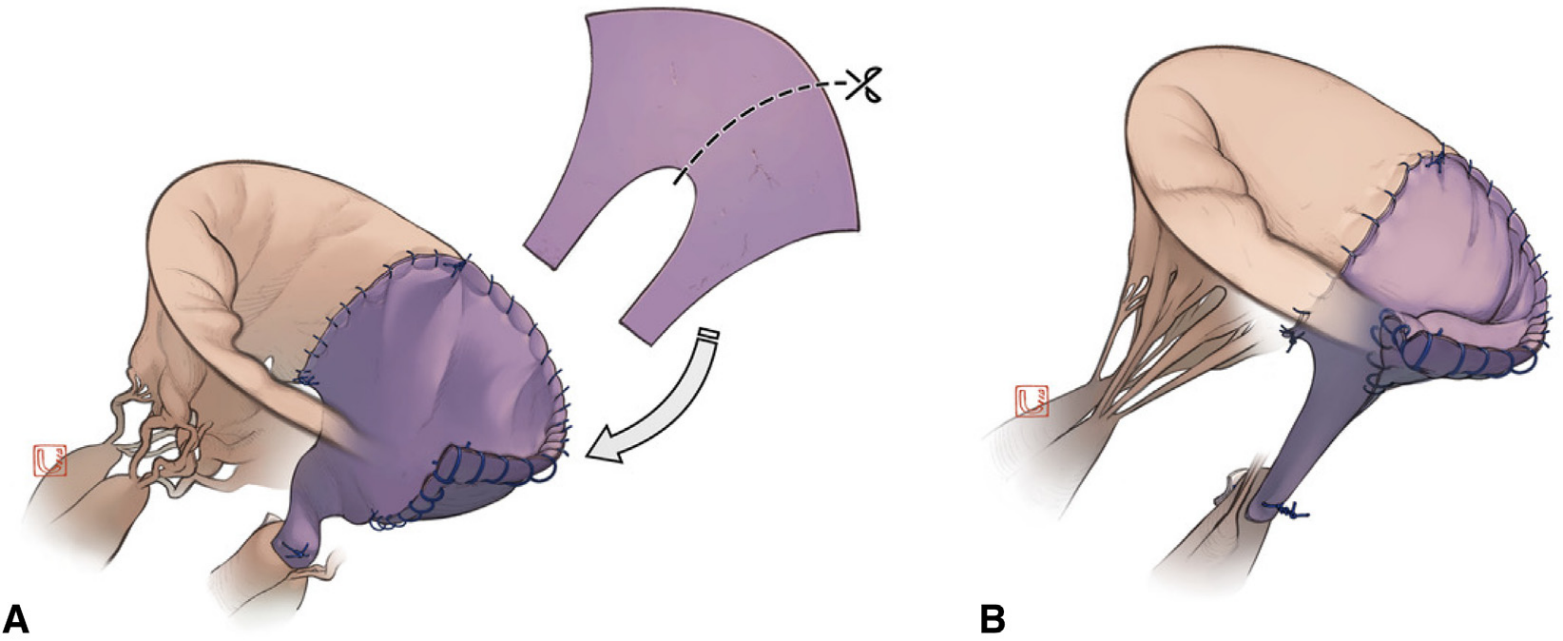
A, Pericardium is secured to the annulus, leaflets, and papillary muscle. B, Good coaptation and commissural structure are created during systole.

Surgery was performed through a full sternotomy or right minithoracotomy. After exposing the MV under cardiac arrest, vegetation, infected leaflets, and chordae were completely removed. When reconstruction involving the commissure was required, partial Normo valve repair was indicated. The surgical procedure is shown in Figure 1 and Video 1. We created a new leaflet and chordae to cover the defect with an autologous pericardium to match approximately half of the larger part of a Normo valve template (Figures 2 and 3). In 4 patients, fresh pericardium was used in the operation. However, in 2 patients, the pericardium was pretreated by soaking in 0.6% glutaraldehyde solution for 10 minutes according to the surgeon's preference for its improved handling characteristics. The trimmed pericardium was sutured to the annulus, remnant leaflets, and papillary muscle. The length of the pericardial leg was determined as the distance between the annulus and papillary muscle. A prosthetic ring annuloplasty was performed in 5 patients with annular dilatation. Indentation closure was added to control residual leakage.

## Discussion

Although the benefits of MV repair for IE are established, the decision to repair or replace depends on the valve damage severity, the patient's condition, and the surgical expertise. Only 33.5% of IE cases are managed with repair because of the complexity of the procedure.^1^

**Figure E1:**
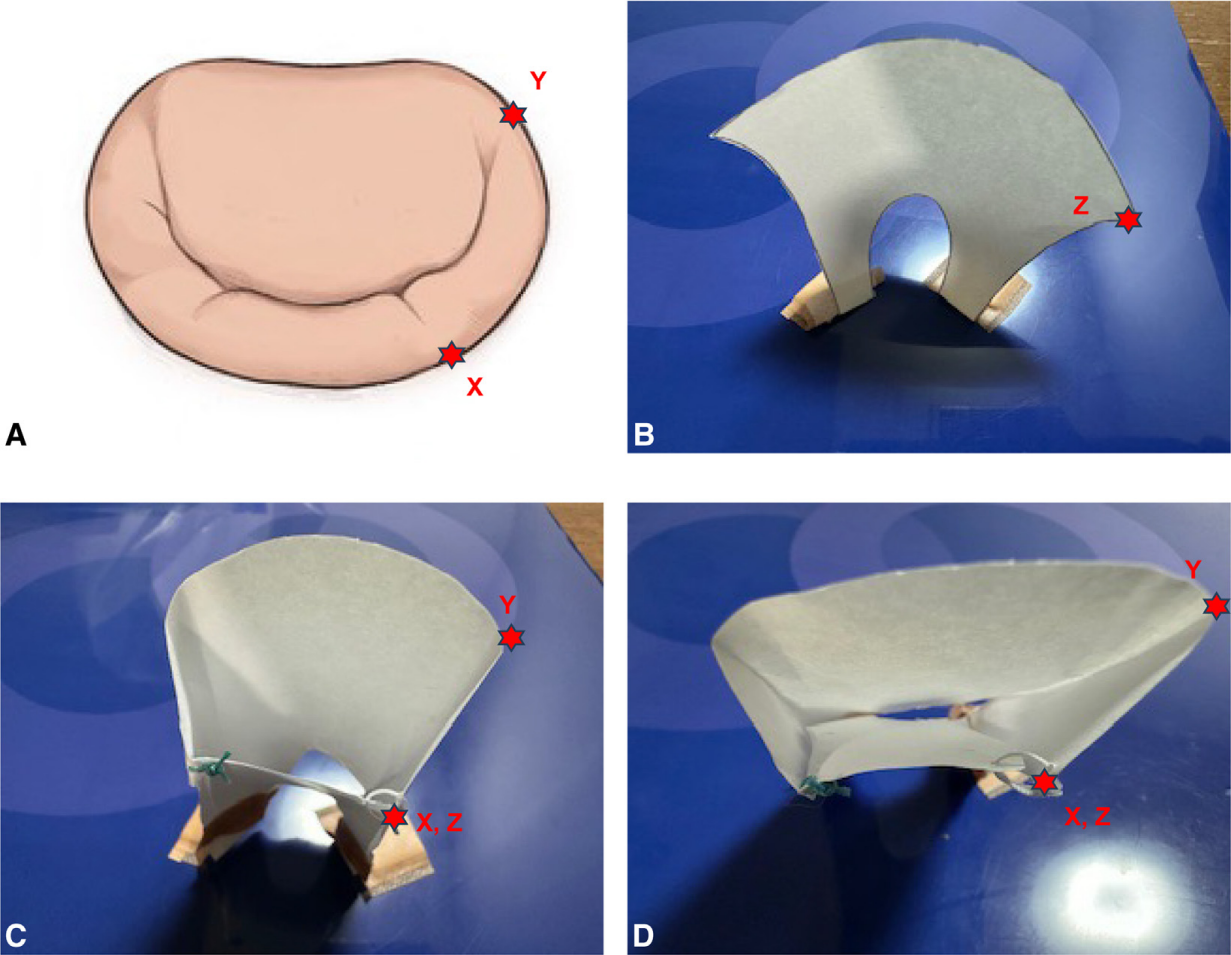
A, MV. B, A larger part of the Normo valve. Z of Normo valve is fixed to X in the MV. C, Normo valve in diastole. D, Folded structure is formed at the commissure in systole.

Okada and colleagues^2^ reported 171 MV surgeries for IE over 20 years. They performed MV repair by combining a basic repair technique for degenerative mitral regurgitation using the pericardium and chordal reconstruction. The rates of 5-year freedom from reoperation were 99% in healed IE and 89.6% in active IE. Early reoperation was required in 4 active cases because of instability of the repair within 1 year. Hosoba and colleagues^5^ reported good long-term outcomes for 49 patients undergoing MV repair using a seamless technique, including 14 with IE.^5^ The rates of 5-year freedom from reoperation were 100%, 92%, and 46% for commissural lesions, 1- to 2-segment involvement, and 3-segment involvement, respectively. Durable leaflets and chordae were reconstructed with a single pericardium covering the commissure or 1 to 2 segments. Our method was applied to 3-segment lesions in 5 patients (Table 1). Half of the larger part of the Normo valve was suitable for reconstruction of 3-segment disease including the commissure. Our method is distinct because of the innovative design of the Normo valve.^2^^,^^3^ Advantages include the simplicity of reconstructing commissural lesions using a unified design (Figures 3 and E1). However, there are some limitations. Although fresh pericardium is preferred because of its suppleness and long-term durability, our series involved fresh and treated pericardium. Additionally, the number of cases was small and follow-up was short. Further research is necessary to validate our method.

## Conclusions

Partial Normo valve repair is a simple and promising technique to reconstruct extensive MV IE involving the commissure.

## Conflict of Interest Statement

The authors reported no conflicts of interest.

The *Journal* policy requires editors and reviewers to disclose conflicts of interest and to decline handling or reviewing manuscripts for which they may have a conflict of interest. The editors and reviewers of this article have no conflicts of interest.

## Supplementary Data

Video 1 Partial Normo valve repair. Video available at: https://www.jtcvs.org/article/S2666-2507(25)00227-5/fulltext.
