# Supplementary figures and images for: Application of stentless mitral valve to surgery in infective endocarditis: Partial Normo valve repair
Source: JTCVS Tech. 2025 May 30;32:72–5. doi: 10.1016/j.xjtc.2025.05.007 (PMC12348339; doi:10.1016/j.xjtc.2025.05.007)

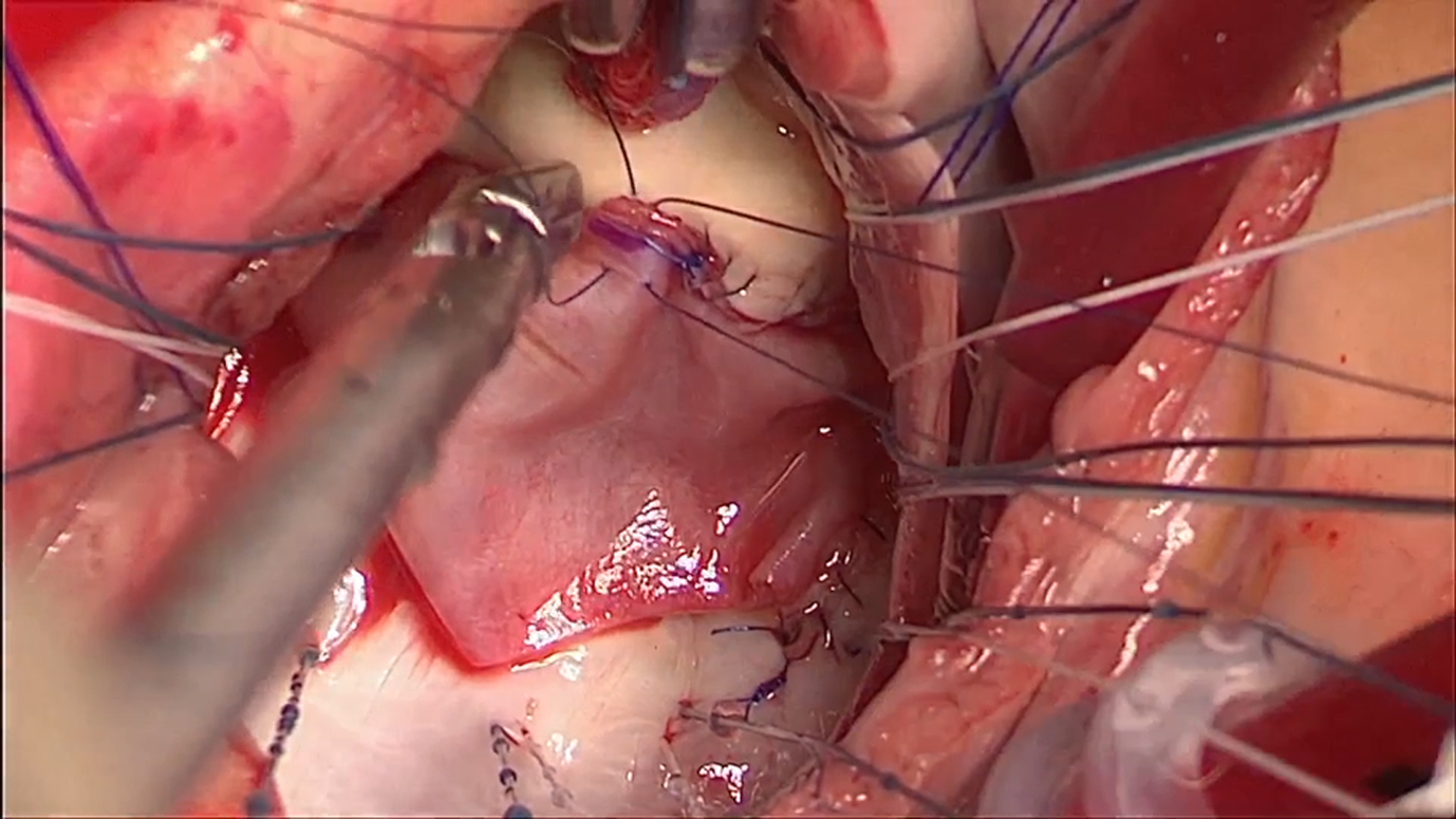

Supplement: Video 1 — Partial Normo valve repair. Video available at: https://www.jtcvs.org/article/S2666-2507(25)00227-5/fulltext. [file fx2.jpg]
